# Liquid-Phase Speed of Sound Measurements of Octafluorocyclobutane (RC-318) and Hexafluoroethane (R-116)

**DOI:** 10.1021/acs.jced.5c00634

**Published:** 2026-01-16

**Authors:** Aaron J. Rowane, Katrina N. Avery

**Affiliations:** Applied Chemicals and Materials Division, National Institute of Standards and Technology, Boulder, Colorado 80305, United States

## Abstract

Sound speed data measured using a dual-path pulse-echo instrument are reported for octafluorocyclobutane (RC-318) at temperatures ranging from 240 to 390 K and at pressures up to 49.01 MPa, and for hexafluoroethane (R-116) at temperatures ranging from 230 to 320 K and at pressures up to 50.27 MPa. The combined expanded uncertainty in the speed of sound varies from 0.035% to 0.102% for RC-318 and 0.039% to 0.097% for R-116 of the measured speed of sound value. The data reported in the present study and those available in the literature are compared to reference equations of state (EOS) for RC-318 and R-116 included in REFPROP version 10.0. The overall performance of both EOS is characterized by an average absolute relative deviation and the maximum deviation. The RC-318 EOS compared to the reported sound speed data with an absolute average relative deviation of 0.56% and a maximum deviation of 1.62%. The R-116 EOS compared to the reported sound speed data with an absolute average relative deviation of 4.85% and a maximum deviation of 6.35%. In both cases, these comparisons show that a refitting of both EOS is required to accurately reproduce the liquid-phase speed of sound for RC-318 and R-116.

## Graphical abstract

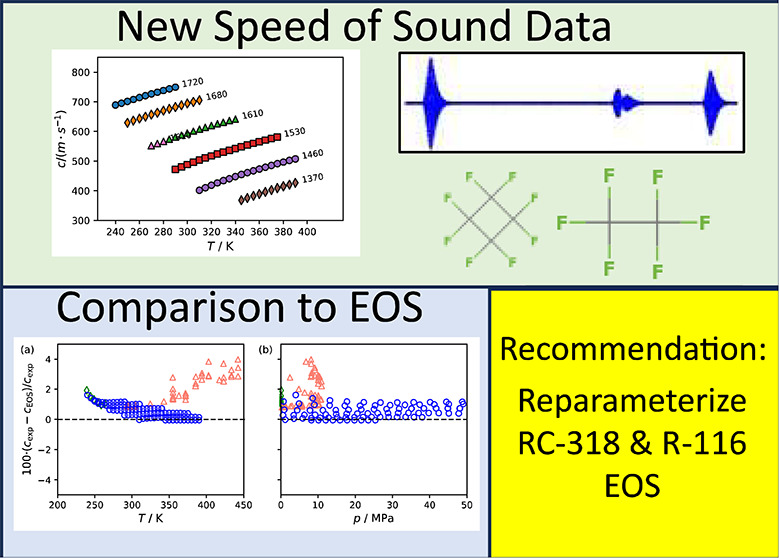

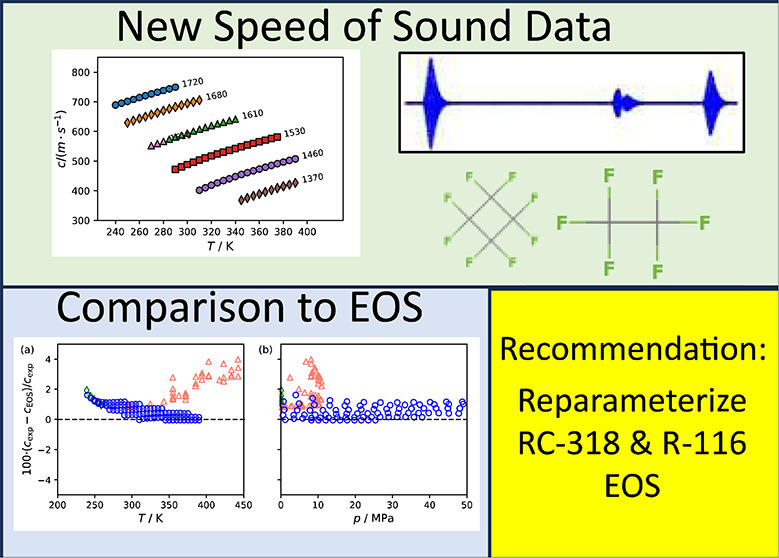

## 1 Introduction

Perfluorocarbons, such as octafluorocyclobutane (RC-318) and hexafluoroethane (R-116) are common etching and cleaning agents used in semiconductor manufacturing processes.^1^ In this study, liquid-phase speed of sound data are reported for RC-318 at temperatures ranging from 240 to 390 K up to a maximum pressure of 49.01 MPa. Liquid-phase speed of sound data for R-116 are reported from 230 to 320 K and up to a maximum pressure of 50.27 MPa. These data were measured in support of ongoing work by the Flow Metering Group at the National Institute of Standards and Technology who are developing a mass flow standard for semiconductor gases.^2,3^ Such mass flow standards require thermophysical property data for their analysis, which are typically calculated using an equation of state (EOS). These mass flow standards can be used to calibrate the mass flow controllers (MFC) used to meter semiconductor gases into process chambers.

Presently, MFCs are calibrated with nitrogen and incorporate empirical correction factors for different process gases. Pope et al.^2^ showed that relative to their mass flow standards the errors in mass flow could be as high as 6% for gases commonly used in semiconductor manufacturing. However, the success of these mass flow standard techniques is contingent on the availability of accurate EOS for various semiconductor gases. The present reference EOS, included in REFPROP version 10.0,^4^ for RC-318 and R-116 are those reported by Platzer et al.^5^ and Lemmon and Span,^6^ respectively. The liquid-phase speed of sound data from the present study are used to test the performance of the present EOS for RC-318 and R-116. If necessary, the speed of sound data reported in this study and other thermophysical property data reported in later studies by our group will be used to develop improved EOS for RC-318 and R-116. While the properties of gases and vapors are of immediate significance to the semiconductor industry; our group is interested in developing EOS capable of representing thermodynamic properties of these fluids over a wide range of conditions in both the liquid and vapor phases.

## 2 Experimental Methods

The following sections describe the samples used in this study and summarize the features of the dual-path pulse-echo instrument used for speed of sound measurements in this study.

### 2.1 Chemical Samples

Table 1 lists the samples used in this study along with their CAS number, normal boiling point, source, and purity. High purity gas samples were obtained directly from the manufacturer circumventing the need for additional degassing or purification. All samples were used as is from the manufacturer.

**Table 1:** Summary of Samples Used in This Work^a^

Chemical	CAS number	Normal Boiling Point (K)	Manufacturer	Analysis Method	Purity[mol/mol]
Octafluorocyclobutane (RC-318)	115–25–3	267.18^4^	Matheson	GC/PDID^b^	0.99999
Hexafluoroethane (R-116)	76–16–4	195.06^4^	General Air	GC/PDID^b^	0.99999

a Both samples were used without further purification. The listed analysis method was performed by the manufacturer of the sample.

b Gas-chromatography/pulse discharge ionization detector.

### 2.2 Dual-Path Pulse-Echo Instrument

Details of the dual-path pulse-echo instrument are described extensively in our previous studies^7,8^ and only the pertinent details are restated here. The dual-path pulse-echo instrument is capable of measurements at temperatures ranging from 228 to 423 K and pressures up to 93 MPa. The standard uncertainties in the temperature and pressure were 0.005 K and 0.016 MPa, respectively. Measurements were performed at a fixed frequency of 8 MHz dictated by the resonant frequency of the quartz ultrasonic transducer incorporated in this instrument. The combined expanded uncertainty in the speed of sound expressed with a coverage factor of *k* = 2 ranged from 0.035% to 0.102% for RC-318 and 0.039% to 0.097% for R-116 of the measured speed of sound value. As discussed in previous studies,^8,9^ the uncertainty in the speed of sound measurements increased as the temperature approached the critical temperature of the sample. The increased uncertainty is due to an increased sensitivity of the speed of sound to a change in pressure and the repeatability of the speed of sound measurement due to weakened echo signals. The details of the uncertainty analysis are described by McLinden and Perkins.^7^

### 2.3 Loading and Isochoric Measurement Procedure

Sample cylinders containing RC-318 and R-116 samples were connected directly to the instrument manifold. Prior to charging the system with sample, the measuring cell, instrument manifold, and filling line were evacuated for at least 12 h at vacuum pressure of 8 × 10^–4^ Pa. The system was then flushed with either RC-318 or R-116. The measuring cell was filled with RC-318 sample at a temperature of 240 K and the R-116 sample was filled at a temperature of 228 K. Once both the measuring cell and manifold were filled with liquid, the first isochore commenced. Measurements for RC-318 started at a temperature of 240 K at a pressure of 4 MPa to avoid solidification of the sample near its triple point temperature of 233.35 K.^4^ The temperature was increased in 5 K increments until the system pressure approached a maximum pressure of 50 MPa or a maximum temperature of 390 K. At the completion of each isochore the system was cooled to the next starting temperature and sample was vented from the system to a pressure roughly 0.5 MPa above its saturation pressure. The first isochore for R-116 started at a temperature of 230 K and the temperature was increased in 5 K increments until a maximum pressure of 50 MPa or maximum temperature of 320 K was reached. Significantly weaker echoes were encountered for the R-116 sample due to its relatively low critical point of 293 K. Therefore, the proceeding isochores were started at temperatures just 5 K higher. Additionally, several higher density isochores were measured by using a hand pump to increase the starting system pressure, which were state points where stronger echo signals were encountered.

## 3 Results and Discussion

The following sections present speed of sound data for RC-318 and R-116. In section 3.2 the speed of sound data reported in this study and those available in the literature are compared to the EOS included in REFPROP version 10.0 for RC-318 and R-116.

### 3.1 Experimental Speed of Sound Data

Figure 1 a and b show the effect of temperature on the speed of sound along pseudoisochores for RC-318 and R-116, respectively. RC-318 measurements were performed from 240 to 390 K to a maximum pressure of 49.01 MPa. R-116 measurements were performed from 230 to 320 K to a maximum pressure of 50.27 MPa. The labels next to each pseudoisochore are densities in [kg·m^–3^] calculated with the corresponding pure fluid EOS, which are averages over the temperature and pressure range of the pseudoisochore rounded to the nearest 10 kg·m^–3^. The measured speed of sound values are listed in Tables 2 and 3 for RC-318 and R-116, respectively. Data listing all the unaveraged speed of sound measurements and their associated uncertainties can be found in the Supporting Information, and these data are also deposited at nist.data.gov (DOI: 10.18434/mds2-3943).

**Figure 1:**
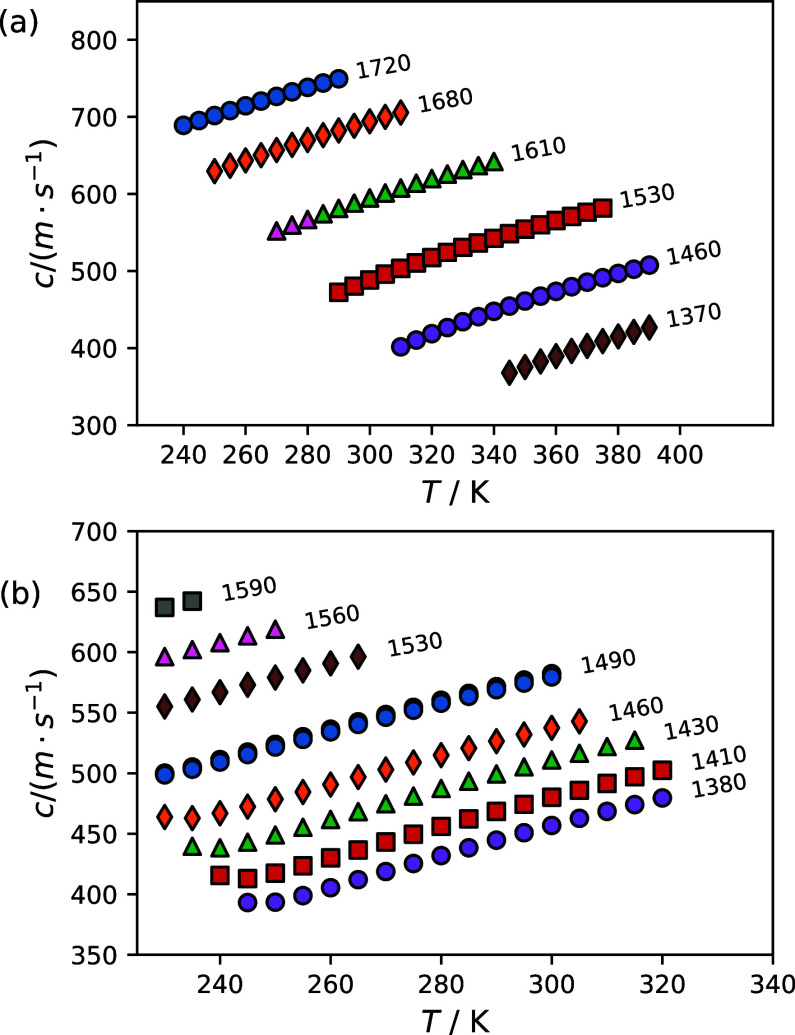
Effect of temperature on the speed of sound along pseudoisochores for (a) octafluorocyclobutane (RC-318), and (b) hexafluoroethane (R-116). Labels are nominal densities for each pseudoisochore in units of [kg·m^–3^], which are the average calculated densities along a pseudoisochore rounded to the nearest 10 kg·m^–3^.

**Table 2:** Experimental Speed of Sound Data for Octafluorocyclobutane (RC-318)^a^

*T*/K	*p*/MPa	*c*/m·s^–1^	100 × *U*_r_(*c*)
239.995	3.989	688.86	0.041
244.994	8.553	695.05	0.040
250.001	13.173	701.58	0.039
254.995	17.739	707.93	0.038
259.996	22.278	714.18	0.038
264.986	26.781	720.34	0.037
269.982	31.261	726.41	0.037
274.976	35.692	732.31	0.036
279.981	40.098	738.11	0.036
284.976	44.460	743.78	0.036
289.982	48.762	749.23	0.035
250.000	0.739	629.42	0.045
254.993	4.899	636.57	0.044
254.994	4.890	636.51	0.044
259.995	9.010	643.37	0.042
264.986	13.112	650.13	0.041
269.982	17.200	656.79	0.040
274.977	21.298	663.48	0.040
279.982	25.328	669.81	0.039
284.977	29.363	676.15	0.038
289.983	33.338	682.21	0.038
294.972	37.301	688.24	0.037
299.974	41.218	694.03	0.037
304.983	45.136	699.83	0.037
309.981	49.012	705.45	0.036
269.981	0.901	551.89	0.054
274.976	4.397	559.60	0.052
279.981	7.849	566.84	0.049
284.976	11.325	574.15	0.048
289.983	14.767	581.11	0.046
294.972	18.199	587.97	0.045
299.973	21.617	594.66	0.044
304.984	25.034	601.23	0.043
309.981	28.420	607.60	0.042
314.981	31.783	613.80	0.041
319.980	35.123	619.85	0.041
324.990	38.447	625.77	0.040
329.983	41.731	631.48	0.040
334.983	44.890	636.59	0.039
339.981	48.096	641.92	0.039
269.981	0.902	551.91	0.054
274.975	4.375	559.46	0.051
279.980	7.832	566.73	0.049
284.975	11.303	574.02	0.048
289.982	14.763	581.09	0.047
294.971	18.177	587.86	0.045
299.972	21.606	594.60	0.044
304.983	25.003	601.07	0.043
309.980	28.385	607.44	0.042
314.980	31.753	613.67	0.041
319.979	35.079	619.66	0.040
324.988	38.403	625.57	0.040
329.982	41.691	631.32	0.039
334.984	44.843	636.41	0.039
339.980	48.057	641.78	0.038
289.980	0.592	472.16	0.071
294.969	3.453	480.34	0.066
299.971	6.322	488.26	0.064
304.981	9.187	495.90	0.061
309.979	12.040	503.29	0.057
314.979	14.885	510.43	0.055
309.979	12.038	503.28	0.056
314.979	14.882	510.42	0.054
319.979	17.716	517.35	0.052
324.988	20.540	524.06	0.050
329.982	23.349	530.60	0.049
334.983	26.031	536.26	0.047
339.980	28.784	542.30	0.046
344.994	31.547	548.30	0.045
350.000	34.285	554.11	0.044
355.010	37.024	559.86	0.043
360.011	39.731	565.41	0.042
365.017	42.425	570.84	0.042
370.015	45.120	576.24	0.041
375.024	47.794	581.48	0.041
309.975	1.157	401.51	0.097
314.977	3.511	410.34	0.089
319.976	5.862	418.60	0.083
324.986	8.218	426.52	0.077
329.980	10.567	434.13	0.073
334.981	12.824	440.70	0.070
339.979	15.138	447.60	0.069
344.992	17.464	454.38	0.069
349.998	19.778	460.94	0.066
355.009	22.087	467.31	0.061
360.010	24.383	473.50	0.056
365.016	26.676	479.54	0.053
365.016	26.677	479.55	0.053
370.015	28.955	485.41	0.052
375.024	31.236	491.19	0.050
380.039	33.497	496.77	0.049
385.047	35.754	502.25	0.048
390.044	37.986	507.55	0.047
344.990	8.202	367.87	0.102
349.997	10.122	375.32	0.098
355.008	12.052	382.59	0.094
360.009	13.967	389.50	0.085
365.015	15.886	396.20	0.085
370.014	17.799	402.69	0.081
375.023	19.711	408.98	0.077
380.039	21.610	415.02	0.070
385.047	23.506	420.90	0.065
390.044	25.390	426.62	0.062

a Speed of sound values listed are averaged from up to 12 measurements at each state point. Isochores are separated by line breaks in order of decreasing density. The standard uncertainties for temperature and pressure are *u*_c_(*T*) = 0.005 K and *u*_c_(*p*) = 0.016 MPa, respectively. Relative combined expanded speed of sound uncertainties *U*_r_(*c*), determined with a coverage factor of *k* = 2, are listed at each state point.

**Table 3:** Experimental Speed of Sound Data for Hexafluoroethane (R-116)^a^

*T*/K	*p*/MPa	*c*/m·s^–1^	100 × *U*_r_(*c*)
230.005	27.581	636.91	0.040
234.997	31.482	642.08	0.039
230.004	20.589	596.29	0.043
234.997	24.301	602.01	0.042
240.000	28.014	607.78	0.041
244.995	31.697	613.45	0.041
250.002	35.337	618.89	0.041
230.004	14.365	555.08	0.054
234.997	17.790	560.83	0.052
240.001	21.255	566.86	0.048
244.995	24.719	572.93	0.046
250.003	28.184	578.97	0.047
254.996	31.621	584.90	0.045
259.997	35.024	590.65	0.044
264.988	38.386	596.20	0.041
230.005	7.367	500.07	0.059
234.997	10.331	505.10	0.056
240.000	13.404	511.07	0.054
240.000	13.421	511.21	0.054
244.995	16.527	517.46	0.052
250.003	19.656	523.83	0.050
254.996	22.774	530.13	0.048
259.996	25.884	536.33	0.047
264.987	28.979	542.43	0.045
269.984	32.069	548.47	0.044
274.977	35.147	554.39	0.043
279.983	38.217	560.23	0.042
284.979	41.260	565.91	0.042
289.984	44.291	571.49	0.041
294.974	47.290	576.92	0.040
299.975	50.267	582.20	0.040
230.005	7.198	498.63	0.059
234.997	10.106	503.25	0.057
240.000	13.162	509.20	0.054
244.995	16.249	515.40	0.055
250.003	19.333	521.57	0.050
254.996	22.435	527.88	0.049
259.997	25.546	534.15	0.047
264.988	28.627	540.26	0.047
269.985	31.690	546.22	0.046
274.978	34.722	552.00	0.044
279.983	37.777	557.83	0.043
284.978	40.804	563.50	0.042
289.984	43.798	568.98	0.041
294.973	46.784	574.40	0.041
299.974	49.704	579.50	0.040
230.004	3.531	463.79	0.070
234.997	5.657	462.85	0.068
240.000	8.243	466.88	0.069
244.995	10.961	472.35	0.062
250.002	13.763	478.49	0.061
254.997	16.529	484.45	0.058
259.996	19.328	490.60	0.054
264.987	22.135	496.79	0.053
269.984	24.950	502.95	0.052
274.978	27.724	508.85	0.049
279.982	30.533	514.88	0.048
284.978	33.300	520.63	0.046
289.984	36.082	526.41	0.045
294.973	38.825	531.96	0.044
299.975	41.563	537.43	0.043
304.984	44.286	542.79	0.042
234.997	3.439	439.60	0.078
240.000	5.404	438.40	0.075
244.995	7.847	442.93	0.072
250.003	10.439	448.93	0.068
254.996	13.068	455.30	0.064
259.997	15.720	461.78	0.061
264.988	18.378	468.25	0.058
269.984	21.047	474.72	0.056
274.978	23.709	481.07	0.054
279.983	26.374	487.32	0.052
284.978	29.014	493.36	0.050
289.984	31.667	499.39	0.049
294.974	34.277	505.14	0.047
299.974	36.902	510.91	0.046
304.985	39.494	516.41	0.045
309.983	42.094	521.95	0.044
314.983	44.662	527.26	0.043
239.999	3.374	415.49	0.088
244.994	5.072	412.90	0.086
250.002	7.332	417.37	0.081
254.996	9.737	423.49	0.076
259.997	12.190	430.01	0.079
264.988	14.653	436.58	0.072
269.984	17.132	443.15	0.069
274.978	19.611	449.62	0.065
279.983	22.097	456.02	0.059
284.978	24.574	462.28	0.056
289.984	27.047	468.41	0.054
294.973	29.496	474.32	0.052
299.975	31.938	480.11	0.050
304.985	34.387	485.86	0.049
309.983	36.826	491.50	0.048
314.983	39.249	497.00	0.046
319.982	41.660	502.39	0.046
244.994	3.483	393.15	0.097
250.002	5.303	393.46	0.094
254.996	7.486	398.87	0.088
259.996	9.766	405.40	0.082
264.988	12.087	412.06	0.077
269.983	14.423	418.74	0.072
274.978	16.768	425.38	0.068
279.983	19.121	431.93	0.065
284.978	21.471	438.37	0.062
289.984	23.822	444.66	0.059
294.973	26.158	450.80	0.057
299.975	28.495	456.82	0.055
304.984	30.824	462.68	0.053
309.982	33.144	468.44	0.051
314.982	35.449	474.04	0.050
319.982	37.751	479.55	0.048

a Speed of sound values listed are averaged from up to 12 measurements at each state point. Isochores are separated by line breaks in order of decreasing density. The standard uncertainties for temperature and pressure are *u*_c_(*T*) = 0.005 K and *u*_c_(*p*) = 0.016 MPa, respectively. Relative combined expanded speed of sound uncertainties *U*_r_(*c*), determined with a coverage factor of *k* = 2, are listed at each state point.

### 3.2 Comparison to REFPROP Version 10.0 Equations of State

In the following sections the experimental speed of sound data reported in this study are compared to speed of sound data for each compound found in the literature relative to reference EOS included in REFPROP version 10.0.^4^

#### 3.2.1 Octafluorocyclobutane (RC-318)

Figure 2 compares experimental speed of sound reported in this study and data available in the literature to the present reference EOS included in REFPROP version 10.0 ^4^ for RC-318. Listed in Table 4 are statistics to summarize the overall performance of the EOS in reproducing the speed of sound values from the present study and those reported in the literature using the absolute average relative deviation (Δ_AARD_), mean deviation bias (Δ_bias_), and the maximum deviation (Δ_MAX_), which are given by,

1 $$\Delta_{\mathrm{AARD}}=100\times \frac{1}{N}\overset{N}{\underset{i=1}{\sum }}\left|\frac{c_{\exp,i}-c_{\mathrm{EOS},i}}{c_{\exp,i}}\right|$$

2 $$\Delta_{\mathrm{bias}}=100\times \frac{1}{N}\overset{N}{\underset{i=1}{\sum }}\frac{c_{\exp,i}-c_{\mathrm{EOS},i}}{c_{\exp,i}}$$

3 $$\Delta_{\mathrm{MAX}}=100\times \max\left(\frac{c_{\exp,i}-c_{\mathrm{EOS},i}}{c_{\exp,i}}\right)$$

respectively, where *N* is the number of data points for a given data set, *c*_exp_,_*i*_ is an experimental speed of sound data point, and *c*_EOS_,_*i*_ is a speed of sound value calculated using an EOS.

**Figure 2:**
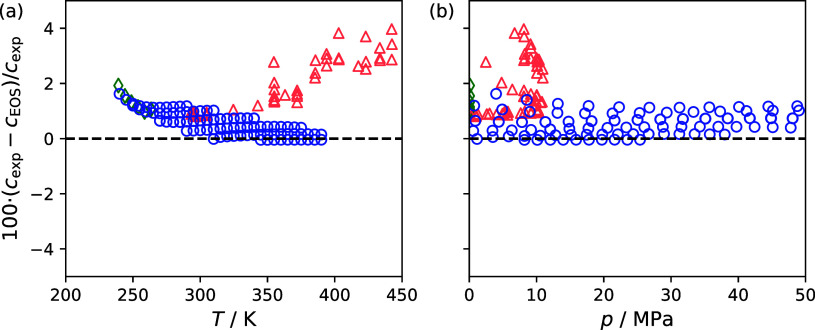
Comparisons of octafluorocyclobutane (RC-318) speed of sound measurements to the equations of state (EOS) of Platzer et al.^5^ as a function of (a) temperature and (b) pressure. Different symbols represent the different studies: This study (○), Meyer^10^ (◊), and Granchenko et al.^11^ (Δ).

**Table 4:** Statistics for Comparison of Data from the Present Study and Those Available in the Literature to the Equation of Platzer et al.^a^ ^5^

Study	Year	*T*_range_/K	*p*_range_/MPa	Δ_AARD_/%	Δ_bias_/%	Δ_MAX_/%
This Study	2025	240–390	0.6–49.1	0.56	0.52	1.62
Granchenko et al.^11^	2019	293–443	0.3–10.9	1.87	1.87	3.97
Meyer^10^	1969	239–264	0.10	1.33	1.33	1.92

a Listed are the citation, year of study, temperature range investigated (*T*_range_), pressure range investigated (*p*_range_), average absolute relative deviation (Δ_AARD_), mean deviation bias (Δ_bias_), and maximum deviation (Δ_MAX_).

The EOS for RC-318 is that of Platzer et al.,^5^ who did not ascribe an uncertainty to speed of sound values calculated with the EOS. The estimated uncertainties of this EOS are 1% for density, 2% for vapor pressure, and 5% for heat capacity. Two literature studies reporting liquid-phase speed of sound data were captured for comparison including that of Meyer^10^ and Granchenko et al.^11^ Figure 2 shows data from the present study compare favorably with the data of Meyer, which ranged from 239.157 to 263.452 K at atmospheric pressure. The data for Granchenko et al. were over a broader temperature range from 293.39 to 442.53 K to a maximum pressure of 10.91 MPa. The data of Granchenko et al. appear to compare favorably with the data from the present study up to a temperature of 340 K, but the data sets appear to conflict at greater temperatures. It is also worth noting that the deviations of Granchenko et al. exhibit significantly more scatter that those reported in the present study.

#### 3.2.2 Hexafluoroethane (R-116)

Figure 3 is a deviation graph that compares experimental speed of sound reported in this study to the present reference EOS included in REFPROP version 10.0 for R-116. The R-116 EOS is that of Lemmon and Span,^6^ who do not assign an uncertainty for speed of sound. Although, Lemmon and Span do assign an uncertainty of 5% for heat capacities, which is a measure of how well an EOS describes derivative properties such as speed of sound. For other properties Lemmon and Span estimate uncertainties of 0.5% for density in both the liquid and vapor phases, 1% for densities in the supercritical state, 0.3% for vapor pressures, and 0.2% for vapor-phase sound speeds. Presently, no other studies report liquid-phase speed of sound data for R-116. As shown in Figure 3 the speed of sound values calculated with EOS of Lemmon and Span deviate by more than 3.56% from the R-116 speed of sound data reported in the present study with a maximum deviation of 6.35%. The overall performance of the EOS in representing the experimental speed of sound data for R-116 is summarized by a Δ_AARD_ of 4.85% and a Δ_bias_ of 4.85%. These significant deviations are unsurprising given that no speed of sound data was fit to the EOS of Lemmon and Span.

**Figure 3:**
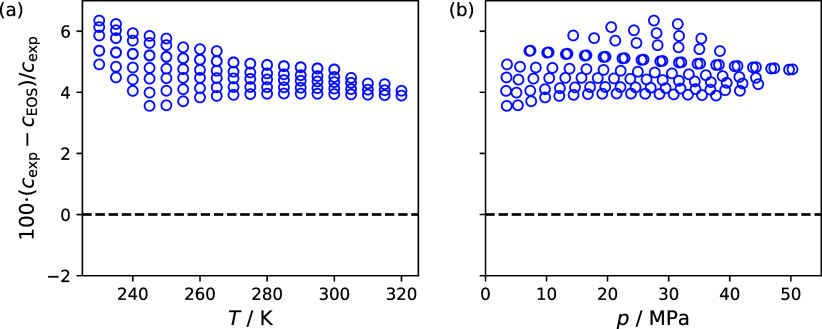
Comparison of hexafluoroethane speed of sound measurements to the equation of state of Lemmon and Span^6^ included in REFPROP version 10.0 ^4^ as a function of (a) temperature and (b) pressure.

## 4 Conclusions

Speed of sound data measured using a dual-path pulse-echo instrument are reported for semiconductor etching and cleaning agents RC-318 and R-116. These data will be used to develop new reference EOS that more accurately represent the thermodynamic properties of RC-318 and R-116. The data from the present study and those in the literature were compared to the present EOS for RC-318 and R-116, which were both found to poorly represent the liquid-phase sound speed. The EOS of Platzer et al.^5^ exhibited a Δ_AARD_ of 0.56% in comparison to the data reported in the present study with deviations ranging from −0.90% to 1.62%. The EOS of Lemmon and Span^6^ for R-116 exhibited a Δ_AARD_ of 4.85% in comparison to the data reported in the present study with deviations ranging from 3.56% to 6.35%. In both cases the deviations between the experimental speed of sound data and speed of sound values calculated with the present REFPROP version 10.0 ^4^ EOS are significantly greater than the estimated uncertainty of the experimental speed of sound data. These deviations are unsurprising given that no liquid-phase speed of sound was used by either Platzer et al. or Lemmon and Span in the development of their EOS for RC-318 or R-116, respectively. In future studies additional property measurements will be performed on both RC-318 and R-116 and the equations of state will be refit to better represent the thermodynamic properties of the fluids.

## Supplementary Material



## Notes

Supporting Information is a ZIP folder containing the unaveraged speed of sound data. The data are also deposited at data.nist.gov (DOI: 10.18434/mds2-3943) (ZIP)
